# Prevalence of comorbidities and concomitant medication use in acromegaly: analysis of real-world data from the United States

**DOI:** 10.1007/s11102-021-01198-5

**Published:** 2022-01-01

**Authors:** Maria Fleseriu, Ariel Barkan, Maria del Pilar Schneider, Yannis Darhi, Amicie de Pierrefeu, Antonio Ribeiro-Oliveira, Stephan Petersenn, Sebastian Neggers, Shlomo Melmed

**Affiliations:** 1 Pituitary Center, Division of Endocrinology, Diabetes and Clinical Nutrition, Departments of Medicine and Neurological Surgery, Oregon Health & Science University, 3303 SW Bond Ave, Mail Code CH8N, Portland, OR 97239 USA; 2 A. Alfred Taubman Health Care Center, University of Michigan, Ann Arbor, MI USA; 3 Ipsen, Les Ulis, France; 4 Ipsen, Boulogne-Billancourt, France; 5 Ipsen, Cambridge, MA USA; 6 ENDOC Center for Endocrine Tumors, Erik-Blumenfeld-Platz 27a, 22587 Hamburg, Germany; 7 Department of Medicine, Section Endocrinology, Pituitary Center Rotterdam, Erasmus University Medical Center Rotterdam, Rotterdam, The Netherlands; 8 Cedars-Sinai Medical Center, Los Angeles, CA USA

**Keywords:** Acromegaly, Comorbidities, Concomitant medications, Injectable medications, Oral medications, Real-world data

## Abstract

**Purpose:**

Patients receiving treatment for acromegaly often experience significant associated comorbidities for which they are prescribed additional medications. We aimed to determine the real-world prevalence of comorbidities and concomitant medications in patients with acromegaly, and to investigate the association between frequency of comorbidities and number of concomitantly prescribed medications.

**Methods:**

Administrative claims data were obtained from the IBM® MarketScan® database for a cohort of patients with acromegaly, identified by relevant diagnosis codes and acromegaly treatments, and a matched control cohort of patients without acromegaly from January 2010 through April 2020. Comorbidities were identified based on relevant claims and assessed for both cohorts.

**Results:**

Overall, 1175 patients with acromegaly and 5875 matched patients without acromegaly were included. Patients with acromegaly had significantly more comorbidities and were prescribed concomitant medications more so than patients without acromegaly. In the acromegaly and control cohorts, respectively, 67.6% and 48.4% of patients had cardiovascular disorders, the most prevalent comorbidities, and 89.0% and 68.3% were prescribed > 3 concomitant medications (p < 0.0001). Hypopituitarism and hypothalamic disorders, sleep apnea, malignant neoplasms and cancer, and arthritis and musculoskeletal disorders were also highly prevalent in the acromegaly cohort. A moderate, positive correlation (Spearman correlation coefficient 0.60) was found between number of comorbidities and number of concomitant medications in the acromegaly cohort.

**Conclusion:**

Compared with patients without acromegaly, patients with acromegaly have significantly more comorbidities and are prescribed significantly more concomitant medications. Physicians should consider the number and type of ongoing medications for individual patients before prescribing additional acromegaly treatments.

**Supplementary Information:**

The online version contains supplementary material available at 10.1007/s11102-021-01198-5.

## Introduction

Acromegaly is generally caused by a growth hormone (GH)-secreting pituitary adenoma, resulting in GH excess and elevated insulin-like growth factor 1 (IGF-1) levels [1–3]. Clinical manifestations of the disease are driven by prolonged GH or IGF-1 exposure, as well as by local tumor compressive effects [4–7]. Typical symptoms of acromegaly include physical manifestations, including enlargement of hands and feet and coarsening of the facial features, as well as arthritis, diabetes, hypertension, and sleep apnea [4, 8, 9]. Excess GH and/or IGF-1 levels also cause metabolic dysfunction and cardiovascular and musculoskeletal comorbidities; these can lead to cardiovascular and respiratory abnormalities and decreased quality of life [4, 10–14]. There is an approximately two-fold excess in mortality in patients with uncontrolled acromegaly compared with the general population [2, 15, 16], as well as an increased cost associated with comorbidity treatment and reduced quality of life [17].

Acromegaly can be treated with surgery, pharmacotherapy [somatostatin receptor ligands (SRLs), GH receptor antagonists, dopamine agonists, or combinations of the above], and/or radiotherapy [2, 18]. In addition to therapies targeting GH and IGF-1 oversecretion, patients often require treatment for acromegaly-related comorbidities, resulting in prescription of multiple medications [19]. Pharmacotherapy treatments for acromegaly and associated comorbidities are administered as oral medications, subcutaneous (SC) injections, and intramuscular (IM) injections [20]. Prescription of multiple medications has been linked with poor adherence and low patient satisfaction and quality of life, especially in patients with chronic illness or more than one comorbidity [21–25], as well as an increased risk of adverse events related to drug–drug interactions [26, 27]. Furthermore, different routes of medication administration are associated with respective advantages and disadvantages related to ease of administration, medication absorption, and reactions with other medications [28, 29]. Taking all these factors into account, it is important for physicians to consider the quantity and form of medications a patient is already receiving when prescribing additional treatments.

Real-world evidence on the frequency of comorbidities and the associated prevalence of prescribed concomitant medications in patients with acromegaly is limited. Accordingly, we determined the prevalence of comorbidities and concomitant medications in a real-world population of patients with acromegaly, as well as in a control cohort of patients without acromegaly, using administrative claims data from the United States (US). Additionally, an analysis of the sub-group of patients with acromegaly receiving prolonged anticoagulant treatment assessed the use of injectable medications in these patients. The sub-analysis was of interest due to the potential risk of bleeding and bruising associated with use of IM injectable medications in conjunction with anticoagulants. This study also sought to evaluate any association between frequency of comorbidities and number of concomitantly prescribed medications in the population of patients receiving anticoagulants.

## Methods

### Study design

This analysis presents results from a real-world, retrospective cohort study of administrative claims data obtained from the IBM® MarketScan® claims database in the US from January 2010 through April 2020. The database is compliant with the US Health Insurance Portability and Accountability Act of 1996 (HIPAA), with all patient-level data de-identified. MarketScan covers resource use and cost data for inpatient and outpatient services, with claims from patients with multiple insurance types throughout the US. Information is derived from medical claims linked to inpatient treatment, outpatient prescription drug claims, and person-level enrollment data.

Claims associated with medical diagnoses and treatment prescriptions were extracted for eligible patients. Comorbidities included in the analysis were selected by referring to published consensus statements and in consultation with the co-authors to reflect acromegaly-related comorbidities seen in clinical practice [15]. An occurrence of a comorbidity was defined by at least one relevant claim. Additionally, the Charlson Comorbidity Index (CCI), a method of categorizing comorbidities based on International Classifications of Diseases (ICD) codes, was calculated for patients with and without acromegaly [30]. In the methodology described by Quan et al. [30], each of 17 pre-specified comorbidity categories have an associated weight based on the adjusted risk of mortality or resource use; the sum of all relevant weights results in a single CCI score for a patient. The use of concomitant medications was defined by at least one relevant claim and the medications were analyzed using the Anatomical Therapeutic Chemical Classification as well as by active ingredient. A sub-analysis of patients with acromegaly receiving prolonged anticoagulant treatment was conducted to investigate the use of injectable medications in these individuals (defined by at least one relevant claim for an injectable medication).

### Patients

A cohort of patients with acromegaly and a control cohort of patients without acromegaly were extracted from the MarketScan database. Patients were eligible for inclusion in the acromegaly cohort if they had: at least two claims associated with any ICD, 9th Revision, Clinical Modification (ICD-9-CM) or ICD, 10th Revision, Clinical Modification (ICD-10-CM) diagnosis codes for acromegaly (E22.0 or 253.0), with more than 30 days between the first and second claim; received at least one treatment for acromegaly with specific treatments including lanreotide depot, octreotide long-acting release (LAR), pasireotide, cabergoline, bromocriptine, and pegvisomant; and had at least 3 months of claims data before the earlier date of either the claim for first diagnosis or the claim for first treatment. Inclusion criteria were selected to ensure that patients had a definite diagnosis of acromegaly, and that the data captured the correct treatment start date, to avoid data bias. As one of the main focuses of this analysis was the use of concomitant medications in patients who were actively receiving medical therapy to treat acromegaly, patients who were not receiving medical therapy (such as those in remission due to surgery and/or radiation) were not included. All patients who met the eligibility criteria for acromegaly using data from MarketScan were included in the study.

Patients were eligible for inclusion in the control cohort if they had no medical claims related to acromegaly, although it was possible for patients in the control cohort to have records for other underlying conditions. A random sample (10%) of patients in the MarketScan database with no medical claims for any ICD-9-CM or ICD-10-CM diagnosis codes for acromegaly (E22.0 or 253.0) within the study period were selected as the initial pool of patients in the control cohort. Direct matching 1:5 (patients with acromegaly to patients without acromegaly) was performed based on age and sex. The observation period (first record date to last record date) of each patient in the control cohort had to include the study period (from the first treatment date to the last record date) of the matched patient with acromegaly. The index date (the date on which the patient fulfilled the eligibility criteria) of each patient in the control cohort was the same as the index date of their matched patient with acromegaly, and in both cohorts, patients had at least 3 months of data prior to the index date.

Patients receiving anticoagulant medications were eligible for inclusion in the anticoagulation sub-analysis if they received an anticoagulant medication (specifically: warfarin, enoxaparin, apixaban, rivaroxaban, fondaparinux, dalteparin, heparin, or dabigatran) daily for > 45 days (to avoid including patients on prophylactic doses of anticoagulants) and had at least one record for an injectable medication (including intravenous [IV], SC, and IM) between the first and last records of an anticoagulant medication. The sub-analysis included four cohorts: patients in the acromegaly cohort receiving anticoagulants; patients in the acromegaly cohort not receiving anticoagulants; patients in the control cohort receiving anticoagulants; and patients in the control cohort not receiving anticoagulants.

### Study outcomes

Outcomes evaluated for the acromegaly and control cohorts included: prevalence of acromegaly-related comorbidities (cardiovascular disorders, arthritis and musculoskeletal disorders, malignant neoplasms and cancer, type 2 diabetes mellitus, sleep apnea, hypopituitarism and disorder of hypothalamus, and bone disorders); the CCI score for each cohort; prevalence and type of prescribed concomitant medications; percentage of patients with each comorbidity receiving 0, 1, 2–3, and > 3 concomitant medications; and correlation between number of comorbidities and number of concomitant medications. Additionally, within the subset of patients who were receiving anticoagulants, the prevalence of comorbidities and prescribed injectable concomitant medications was analyzed.

### Statistical analysis

Demographic characteristics were analyzed using descriptive statistics, with continuous variables presented as the mean and standard deviation (SD), and categorical or discrete variables presented as percentages. Python language was used for data preparation and statistical analyses [31]. Chi-squared tests were used for inter-cohort proportional comparisons and mean CCI score was compared between the acromegaly and control cohorts using an unpaired t-test. An unpaired t-test was also used to compare the average count of different active medication ingredients between the acromegaly and control cohorts. Medications containing multiple active ingredients were analyzed by individual ingredient. Spearman correlation coefficients and p-values were calculated between the number of different concomitant medications and the number of different comorbidities. For all analyses, a p-value of < 0.05 was considered statistically significant.

## Results

### Patient disposition and baseline characteristics

The acromegaly cohort included 1175 patients, while the matched control cohort included 5875 patients; in both cohorts, 50.1% of patients were female and mean (SD) age was 48.5 (14.0) years. Complete demographics and baseline characteristics are presented in Table 1. A total of 52 patients were included in the sub-cohort of patients with acromegaly receiving anticoagulants; 1123 patients were included in the sub-cohort of patients with acromegaly not receiving anticoagulants; 131 patients without acromegaly were included in the control cohort of patients receiving anticoagulants; and 5744 patients without acromegaly were included in the control cohort of patients not receiving anticoagulants. Complete demographics and baseline characteristics for the anticoagulation sub-analysis are presented in Table 2.

**Table 1:** Demographics and baseline characteristics in the acromegaly and control cohorts

	Acromegaly cohort (N = 1175)	Control cohort (N = 5875)
Sex, n (%)		
Male	586 (49.9)	2930 (49.9)
Female	589 (50.1)	2945 (50.1)
Age		
Mean (SD)	48.5 (14.0)	48.5 (14.0)
Median (95% CI)	50.0 (49.0–51.0)	50.0 (49.0–51.0)
Study period (index date to end of data) (years)		
Mean (SD)	2.8 (2.4)	2.8 (2.4)
Median (95% CI)	2.1 (1.9–2.2)	2.1 (1.9–2.2)

*CI* confidence interval, *SD* standard deviation

**Table 2:** Demographic characteristics for the sub‑analysis of patients with acromegaly receiving anticoagulants

	Case cohort	Comparison groups		
	Patients with acromegaly receiving anticoagulants (N = 52)	Patients with acromegaly not receiving anticoagulants (N = 1123)	Patients without acromegaly receiving anticoagulants (N = 131)	Patients without acromegaly not receiving anticoagulants (N = 5744)
Sex, n (%)				
Male	33 (63.5)	553 (49.2)	71 (54.2)	2859 (49.8)
Female	19 (36.5)	570 (50.8)	60 (45.8)	2885 (50.2)
Age				
Mean (years)	60.4	47.8	62.0	48.2

### Prevalence of comorbidities

**Fig. 1:**
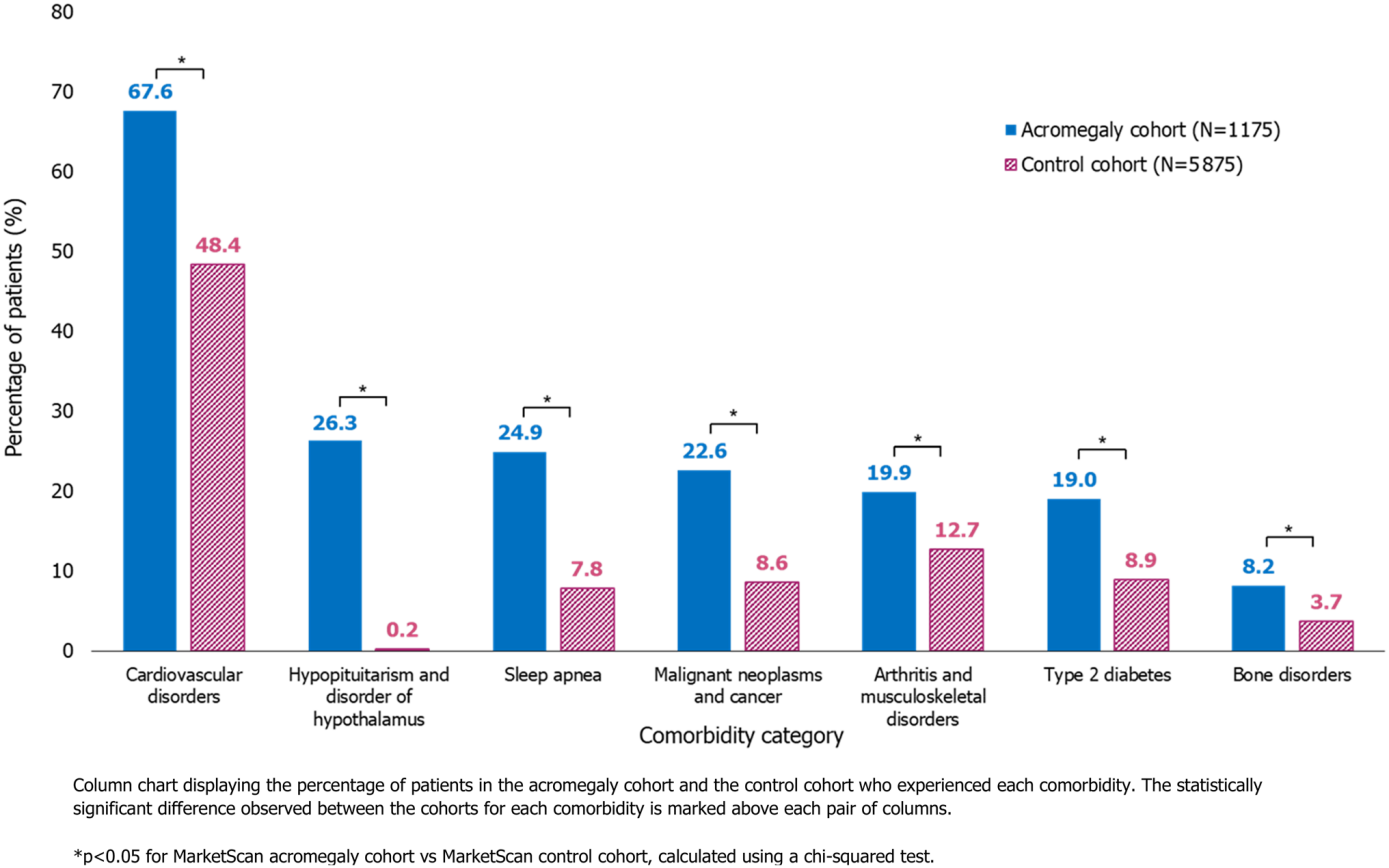
Prevalence of comorbidities in the acromegaly and control cohorts

Cardiovascular disorders were the most prevalent comorbidity category observed across both the acromegaly and control cohorts, experienced by 67.6% and 48.4% of patients, respectively. In the acromegaly versus the control cohort, there was also a higher prevalence of hypopituitarism and disorders of hypothalamus (26.3% vs. 0.2%), sleep apnea (24.9% vs. 7.8%), malignant neoplasms and cancer (22.6% vs. 8.6%), and arthritis and musculoskeletal disorders (19.9% vs. 12.9%, respectively; Fig. 1). Compared with patients in the control cohort, a significantly higher percentage of patients in the acromegaly cohort experienced each comorbidity category (each p < 0.05). Similarly, the mean CCI score was significantly higher in the acromegaly cohort (1.4) than in the control cohort (0.58; p < 0.0001; Table 3).

**Table 3:** Charlson comorbidity index of patients in the acromegaly and control cohorts

	Acromegaly cohort (N = 1175)	Control cohort (N = 5875)
CCI		
Mean*	1.4	0.58
Median	1.0	0.0
SD	2.1	1.3
95% CI	(1.2–1.5)	(0.55–0.62)

*CCI* Charlson Comorbidity Index, *CI* confidence interval, *SD* standard deviation

*p-value < 0.0001 for acromegaly cohort vs. control cohort, calculated using an unpaired t-test

In the sub-analysis of patients receiving prolonged anticoagulant treatment, cardiovascular disorders were the most prevalent comorbidities in all cohorts, reported in 98.1%, 66.6%, 93.9%, and 46.6% of patients with acromegaly receiving anticoagulants, patients with acromegaly not receiving anticoagulants, patients in the control cohort receiving anticoagulants, and patients in the control cohort not receiving anticoagulants, respectively. Patients with acromegaly receiving anticoagulants had a significantly higher prevalence of type 2 diabetes mellitus and glucose intolerance and malignant neoplastic disease than all three comparison cohorts (diabetes: 48.1% vs. 19.1%, 29.0%, and 8.1% of patients, respectively; malignant neoplastic disease: 48.1% vs. 21.6%, 0.0%, and 0.0% of patients, respectively) and experienced a significantly higher prevalence of all comorbidities when compared with the cohort of patients without acromegaly not receiving anticoagulants (p < 0.05; Table 4).

**Table 4:** Prevalence of comorbidities in all cohorts of the anticoagulation sub-analysis

	Patients with acromegaly receiving anticoagulants (N = 52)	Patients with acromegaly not receiving anticoagulants (N = 1123)	Patients without acromegaly receiving anticoagulants (N = 131)	Patients without acromegaly not receiving anticoagulants (N = 5744)
Comorbidity, n (%)				
Cardiovascular disorders	51 (98.1)	748 (66.6)*	123 (93.9)	2676 (46.6)*
Malignant neoplasms and cancer	25 (48.1)	243 (21.6)*	0 (0.0)*	2 (0.03)*
Type 2 diabetes mellitus and glucose intolerance	25 (48.1)	215 (19.1)*	38 (29.0)*	466 (8.1)*
Sleep apnea	20 (38.5)	274 (24.4)*	39 (29.8)	387 (6.7)*
Hypopituitarism and disorder of hypothalamus	15 (28.8)	297 (26.4)	0 (0.0)*	8 (0.14)*
Arthritis and musculoskeletal disorders	15 (28.8)	225 (20.0)	34 (26.0)	697 (12.1)*
Bone disorders	6 (11.5)	90 (8.0)	18 (13.7)	166 (2.8)*

*p < 0.05 for comparison with the cohort of patients with acromegaly receiving anticoagulants, calculated using a chi-squared test

### Prescription of concomitant medications

**Fig. 2:**
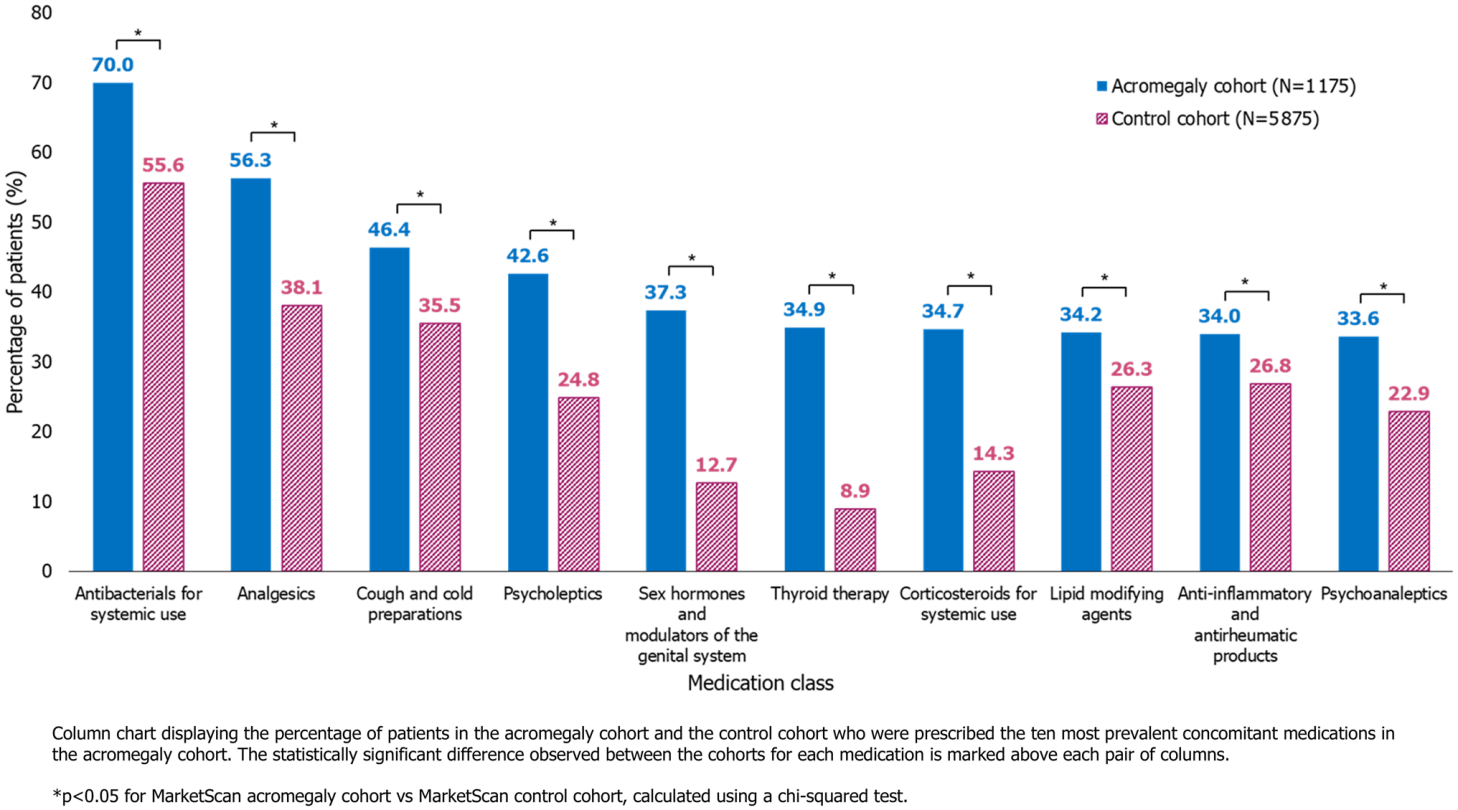
Prevalence of concomitant medications by Anatomical Therapeutic Chemical Classification in the acromegaly and control cohorts

A significantly higher percentage of patients in the acromegaly cohort than in the control cohort were prescribed each class of concomitant medication (each p < 0.05; Supplementary Table 1). The most prevalent class of concomitant medications for both cohorts was antibacterials for systemic use (70.0% vs. 55.6%, respectively), followed by analgesics (56.3% vs. 38.1%) and cough and cold preparations (46.4% vs. 35.5%). A high percentage of the acromegaly cohort were additionally prescribed psycholeptics (42.6%) and sex hormones and modulators of the genital system (37.3%); these medication classes were less prevalent in the control cohort, prescribed to 24.8% and 12.7% of patients, respectively (Fig. 2).

Most concomitant medications were taken in oral form (67.9%), 15.1% were taken as injectables, and 17.0% were taken in a form other than oral or injectable. The analysis found that regardless of route of administration, all concomitant medication ingredients except for lisinopril (an oral medication) were prescribed to significantly more patients in the acromegaly cohort than the control cohort (p < 0.05; Supplementary Table 2).

**Fig. 3:**
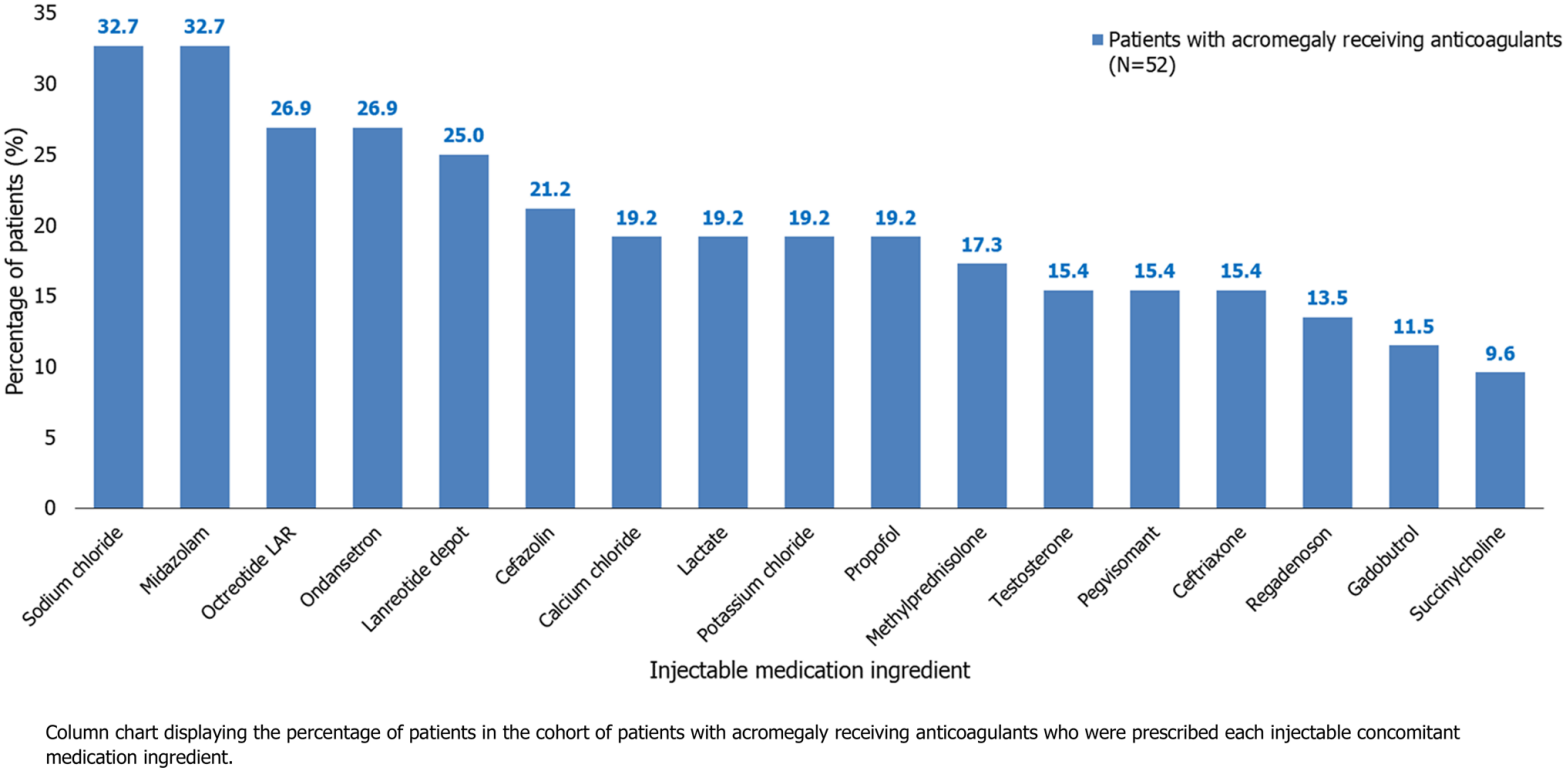
Injectable medications by ingredient in the cohort of patients with acromegaly receiving anticoagulants

For patients receiving anticoagulants, a significantly higher percentage of patients with acromegaly were prescribed concomitant injectable medications compared with those without acromegaly (88.5% vs. 67.9%; p = 0.0078). The injectable medication ingredients most frequently prescribed to patients with acromegaly who were receiving anticoagulants were: IV sodium chloride (32.7% of patients), IM/IV midazolam (32.7%), IM octreotide LAR (26.9%), IV ondansetron (26.9%), SC lanreotide depot (25.0%), and IM/IV cefazolin (21.2%; Fig. 3).

### Number of prescribed concomitant medication ingredients by comorbidity

Across the acromegaly and control cohorts, respectively, 2.5% and 15.0% of patients were prescribed 0 concomitant medication ingredients (p < 0.0001), 3.1% and 5.4% were prescribed 1 (p = 0.0014), 5.4% and 11.3% were prescribed 2–3 (p < 0.0001), and 89.0% and 68.3% were prescribed > 3 (p < 0.0001), accounting for medications taken via all routes of administration (oral, injectable, and neither oral nor injectable).

**Fig. 4:**
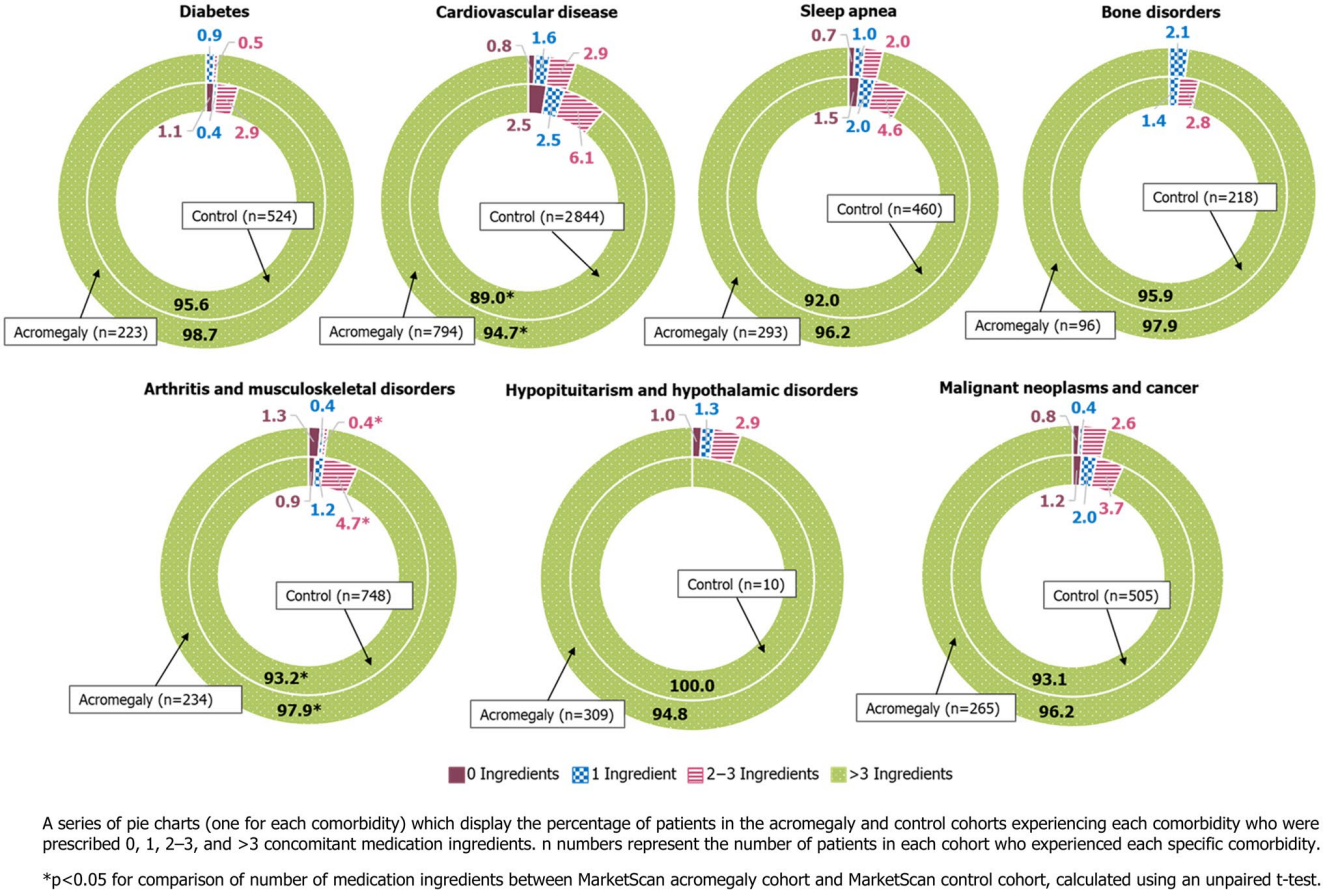
Percentage of prescribed concomitant medication ingredients by comorbidity in the acromegaly and control cohorts

Usage of concomitant medications in patients with comorbidities was high, regardless of acromegaly diagnosis, with more than 85% of patients being prescribed > 3 concomitant medication ingredients (Fig. 4). When analyzed by route of administration, this high concomitant usage was driven by oral medications more than injectable medications (Supplementary Table 3). For any given comorbidity, over 80% of patients were prescribed > 3 oral medications; for injectable medications this was fewer than 52% of patients.

### Correlation analysis

**Fig. 5:**
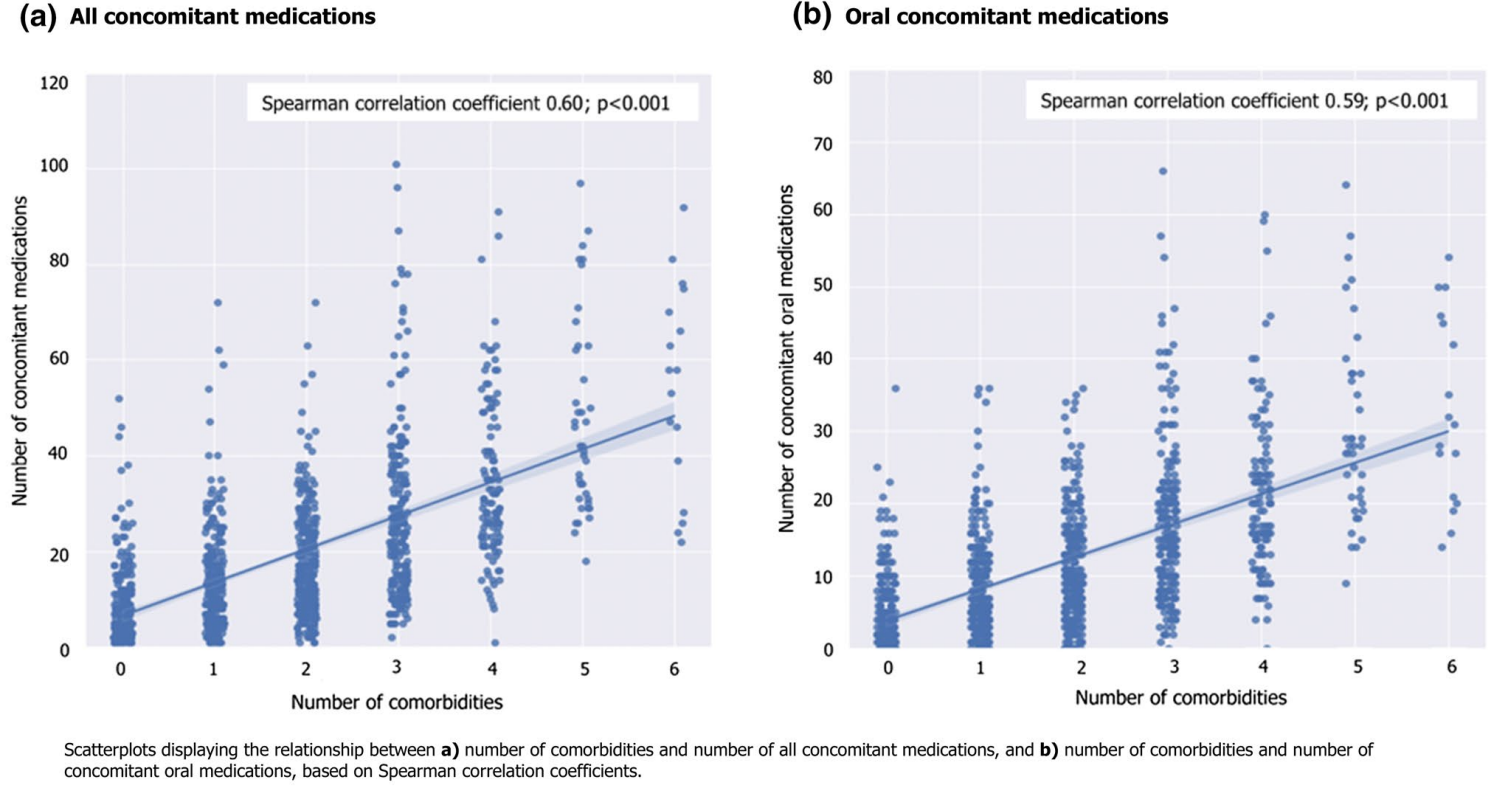
Correlation between number of comorbidities and number of prescribed concomitant medications in the acromegaly cohort

Moderate positive associations were found between the number of comorbidities and number of all concomitant medications (including medications of all reported routes of administration; oral, injectable, and neither oral nor injectable) and oral concomitant medications, indicated by Spearman correlation coefficients of 0.60 and 0.59, respectively (both p < 0.001; Fig. 5). Conversely, low positive correlations were reported between number of comorbidities and number of injectable and neither oral nor injectable concomitant medications, as indicated by a Spearman correlation coefficient of 0.45 for each (both p < 0.001).

## Discussion

In this real-world, controlled analysis, patients with acromegaly had a higher frequency of acromegaly-related comorbidities and were prescribed more concomitant medications compared with patients without acromegaly. The prevalence of comorbidities in our study generally aligned with the incidence rates of comorbidities previously reported. In a real-world study in the US assessing 23 acromegaly-related comorbidities, high incidence rates for arthropathy, arthralgia, synovitis, hypertension, hypopituitarism, osteoarthritis, and diabetes were reported [19]. An observational study showed that the most prevalent comorbidities were endocrine and metabolic diseases, including diabetes, bone diseases, and hypopituitarism (96.7%); cardiovascular diseases (70.7%); and musculoskeletal disorders (22.0%) [32]. Similarly, a large study using the Liege Acromegaly Survey Database reported high rates of metabolic disorders (such as diabetes), cardiovascular disorders, and thyroid disorders in patients with acromegaly [33]. As expected, each acromegaly-related comorbidity observed in our study was experienced by significantly more patients with acromegaly compared with the control cohort. For instance, the proportion of patients with cerebrovascular disorders (within the cardiovascular disorders category) in the acromegaly cohort was nearly twice that of the matched control cohort. Additionally, the significantly higher CCI score for patients with acromegaly calculated in our analysis demonstrates a broad comorbidity presence. While the CCI has not been validated for acromegaly specifically, it provides a valuable, quantified measurement for comparison between the acromegaly and control cohorts. While it would have been interesting to explore the interaction between different comorbidities in the anticoagulation sub-analysis, the descriptive nature of the analysis and the small number of patients with acromegaly receiving anticoagulants precluded the feasibility of conducting a multivariate analysis.

The high prevalence of comorbidities in patients with acromegaly was reflected in the observed frequency of prescribed concomitant medications. For example, 19.0% of patients in the acromegaly cohort had a diagnosis of diabetes and 24.4% of patients were receiving diabetes medications. The correlation analysis further suggested a link between comorbidities and concomitant medication usage, with a moderate, positive association found between the number of comorbidities and the number of all and oral concomitant medications. This result confirms that patients with acromegaly experience an increased frequency of acromegaly-related comorbidities, which may in turn require a greater number of concomitant medications. Among the concomitant medications prescribed to a significantly greater number of patients with acromegaly were psychotropic and pain medications, underscoring the unmet need for a focus on mental health and pain management required for patients with acromegaly.

In aggregate, we report significantly higher concomitant medication use in patients with acromegaly than in patients without acromegaly (89.0% and 68.3% of the acromegaly and control cohorts, respectively, received > 3 concomitant medications). However, in both the acromegaly and control cohorts, most patients with comorbidities were prescribed > 3 concomitant medications. This finding confirms our expectation that increased concomitant medication usage is driven by comorbidities. Due to the higher overall prevalence of comorbidities in patients with acromegaly, these patients are also prescribed more concomitant medications. A prior study reported that approximately 60% of all adults in the US were prescribed ≥ 1 medication, with 15% reporting the use of ≥ 5 prescription medications [34]. In our study, 97.5% of patients with and 85.0% of patients without acromegaly were prescribed ≥ 1 medication; conversely, only 2.5% of patients with acromegaly were prescribed 0 concomitant medications, versus 15.0% of patients without acromegaly. These results suggest that while patients without acromegaly are often prescribed multiple medications, the overall prevalence of concomitant medications is substantially higher in patients with diseases such as acromegaly, and it is uncommon for a patient receiving acromegaly treatment to be prescribed no additional medications.

Almost all medications, regardless of route of administration, were observed to be more frequently prescribed for patients with acromegaly. The one exception, lisinopril, is an oral medication used to treat cardiovascular disease. The high prevalence of cardiovascular disorders in patients with and without acromegaly likely accounts for the comparable usage of lisinopril in both cohorts. Notably, the majority of all concomitant medications were oral medications. Physicians should consider the types of ongoing medications for individual patients during treatment decision-making, as the most appropriate treatment option may need to be adjusted depending on the number and type of medications that a patient is already receiving. Challenges posed by the prescription of multiple oral medications include managing potential drug–drug interactions as well as abiding by any food restrictions [26, 27, 35]. For example, many of the oral medications used to treat thyroid disorders and bone diseases (prescribed to 35.4% and 2.7% of patients with acromegaly in our analysis, respectively), as well as certain drugs to treat diabetes, are commonly taken while fasting [36–38]. Patients taking levothyroxine or alendronate must also generally continue fasting for a period of time before taking any other drugs [37, 38], including those to treat acromegaly. Furthermore, proton-pump inhibitors, prescribed in oral form for 22.2% of patients with acromegaly in this study, alter the acidic environment of the stomach and thus can impact absorption of other oral medications [39, 40].

On the other hand, surveys and clinical trials have shown that some patients experience pain with injections, fear of injections, or injection-site reactions such as nodules or indurations, and may therefore prefer to take oral medications [41–43]. Additionally, IM injectable medications in particular may pose other potential risks and complications not evident with oral or SC routes of administration when administered alongside anticoagulants. The sub-analysis found that patients with acromegaly were more likely to be receiving anticoagulants and more likely to receive injectable concomitant medications than those without acromegaly. These injectable medications include both SC and IM injections (such as IM depot testosterone). Certain studies have suggested that SC injections are preferable to IM injections due to fewer side effects (such as injection-site pain), and may allow for lower doses [44]. Furthermore, United Kingdom (UK) guidelines recommend against the use of IM injections in patients receiving anticoagulation therapy due to the risk of developing hematomas and bleeding [45]; this risk is not present for SC injections. While limited data from UK guidelines suggest that IM injections may be used safely with newer oral anticoagulants such as dabigatran, rivaroxaban, edoxaban, or apixaban when administered 24 hours after previous anticoagulant dose [45], IM injections may not be a viable option for patients who are prescribed anticoagulants for daily use. Notably, the potential risk posed by IM injections for people receiving anticoagulants is not mentioned in US cardiology guidelines [46–48], which may in part explain the prevalence of IM injectable medications observed in the anticoagulation sub-analysis.

In order to alleviate the potential medication burden on patients and to encourage medication adherence, it is important to consider patient preference as well as the varying levels of risk or complications associated with different modes of administration when prescribing treatments for acromegaly. For instance, in cases where patients are already receiving a complex regimen of oral medications that requires fasting, it may be preferable to prescribe additional medications in injectable form wherever possible, while an oral or SC route of administration for additional medications may be preferred for patients treated with anticoagulants.

Clinical trials in acromegaly reported similar findings to this real-world analysis. In the PRIMARYS (NCT00690898) and LEAD (NCT00701363) open-label trials investigating lanreotide depot, patients with acromegaly generally experienced a high prevalence of comorbidities, and most patients were prescribed one or more concomitant medications [49]. While results from clinical trials are generally robust and well-controlled, data derived from claims databases cover more diverse patient populations and may provide a more accurate representation of patients in the real-world and their clinical management.

### Strengths

To our knowledge, this study is the first to clearly define comorbidities associated with acromegaly compared with a control population without acromegaly, and to investigate a wide range of associated concomitant medications in a real-world setting. Previous studies have assessed concomitant medication use in patients with acromegaly, generally focusing on specific classes of medications [50]. The claims database used in this study is large, containing thousands of patients with acromegaly and up-to-date results spanning approximately the last decade. The availability of data for patients without acromegaly allowed for a matched control cohort for comparison with the acromegaly cohort. Finally, 1:5 matching reduced the standard error of the outcome variables in the control group, therefore strengthening the power of the between-group comparisons.

### Limitations

This study analyzed data solely from the US; as such, generalization to other populations is limited. Since the results were obtained from an administrative claims database, missing data or coding errors were encountered, and it was not possible to verify actual rates of medication adherence. In addition, laboratory data to determine biochemical control of acromegaly, such as GH and IGF-1 levels, were not available. It should be noted that concomitant medication prescriptions were analyzed throughout the course of each patient’s study period. While this method ensured that all prescriptions were counted, patients may have received different prescriptions at different times, and therefore may not have been taking all recorded medications at the same time. Furthermore, as this analysis sought to explore concomitant medication use in patients receiving medical therapy for acromegaly, patients in remission due to surgery or radiotherapy would not have been included. Nevertheless, patients with acromegaly were prescribed significantly more medications than patients without acromegaly over the course of the study. While analysis of concomitant medications evaluated all concomitant medications used in patients with acromegaly, the comorbidities analysis was limited to comorbidities known to be prevalent in patients with acromegaly. The CCI score helped mitigate this limitation by accounting for a broader array of comorbidities. It is also possible that patients with acromegaly are monitored more closely than patients without acromegaly, which could lead to over-reporting of comorbidities in the acromegaly cohort. As this was a descriptive study, no causal associations between patients with acromegaly and outcomes of interest could be determined using the real-world data. This study also included no adjustment for multiplicity/hypothesis generation.

## Conclusions

This analysis highlighted the high prevalence of comorbidities and frequency of prescribed concomitant medications in patients with acromegaly. Patients with acromegaly had significantly more comorbidities than patients without acromegaly and were prescribed significantly more concomitant medications for all medication classes and almost all medication ingredients, including those taken in oral and injectable forms, and in forms other than oral or injectable. Most patients in the acromegaly cohort were prescribed > 3 concomitant medications, and the number of comorbidities and number of prescribed concomitant medications were moderately positively correlated. Additionally, patients with acromegaly were more likely to be receiving anticoagulants and concomitant injectable medications (including IM injections) than patients without acromegaly. Use of real-word data enables the exploration of a heterogenous population beyond clinical trials, to gain insights into current medical practices and treatments. Physicians should consider the frequency and form of patients’ existing concomitant medications when prescribing treatments for acromegaly, particularly given the availability of treatments with different methods of administration.

## Supplementary Information

Below is the link to the electronic supplementary material.

Supplementary file1 (PDF 153 kb)

## Data Availability

Owing to the nature of this claims database analysis, Ipsen does not have access to individual participant data. The datasets analyzed in this study are available through the IBM® MarketScan® database. However, the authors cannot share the data with any third parties or make the data publicly available owing to protections around the sharing of private health data.
