# Supplementary material for: Prevalence of comorbidities and concomitant medication use in acromegaly: analysis of real-world data from the United States
Source: Pituitary. 2022 Jan 1;25(2):296–307. doi: 10.1007/s11102-021-01198-5 (PMC8894179; doi:10.1007/s11102-021-01198-5)
Supplement: Supplementary file 1 — Supplementary file1 (PDF 153 kb) [file 11102_2021_1198_MOESM1_ESM.pdf]

SUPPLEMENTARY DATA

Prevalence of comorbidities and concomitant medication use in acromegaly: Analysis of real-world data from the United States

Journal: *Pituitary*

Maria Fleseriu, MD,[1] Ariel Barkan, MD,[2] Maria del Pilar Schneider, PhD,[3] Yannis Darhi, MSc,[4] Amicie de Pierrefeu, PhD,[4] Antonio Ribeiro-Oliveira Jr, MD, PhD,[5] Stephan Petersenn, MD,[6] Sebastian Neggers, MD,[7] Shlomo Melmed, MD[8]

[1]Pituitary Center at Oregon Health & Science University, Portland, OR, USA; [2]A. Alfred Taubman Health Care Center, University of Michigan, MI, USA; [3]Ipsen, Les Ulis, France; [4]Ipsen, Boulogne-Billancourt, France; [5]Ipsen, Cambridge, MA, USA; [6]ENDOC Center for Endocrine Tumors, Erik-Blumenfeld-Platz 27a, 22587 Hamburg, Germany; [7]Department of Medicine, Section Endocrinology, Pituitary Center Rotterdam, Erasmus University Medical Center Rotterdam, Rotterdam, The Netherlands; [8]Cedars-Sinai Medical Center, CA, USA

Correspondence to:

Maria Fleseriu, MD

Pituitary Center, Departments of Medicine (Division of Endocrinology, Diabetes and Clinical Nutrition) and Neurological Surgery,
Oregon Health & Science University, Portland, Oregon, USA

Address correspondence to:
Maria Fleseriu, MD
Oregon Health & Science University
Mail Code CH8N
3303 SW Bond Ave
Portland, Oregon 97239
USA

Email: fleseriu@ohsu.edu
ORCID https://orcid.org/0000-0001-9284-6289

Supplementary Table 1 Prevalence of all concomitant medications by anatomic therapeutic class

	Acromegaly cohort (N=1,175)	Control cohort (N=5,875)	
**Anatomical Therapeutic Chemical classification, n (% of patients)**			**p-value**
Antibacterials for systemic use	822 (70.0)	3267 (55.6)	<0.0001
Analgesics	661 (56.3)	2239 (38.1)	<0.0001
Cough and cold preparations	545 (46.4)	2084 (35.5)	<0.0001
Psycholeptics	501 (42.6)	1455 (24.8)	<0.0001
Sex hormones and modulators of the genital system	438 (37.3)	746 (12.7)	<0.0001
Thyroid therapy	410 (34.9)	520 (8.9)	<0.0001
Corticosteroids for systemic use	408 (34.7)	842 (14.3)	<0.0001
Lipid modifying agents	402 (34.2)	1547 (26.3)	<0.0001
Anti-inflammatory and antirheumatic products	400 (34.0)	1575 (26.8)	<0.0001
Psychoanaleptics	395 (33.6)	1343 (22.9)	<0.0001
Agents acting on the renin-angiotensin system	391 (33.3)	1440 (24.5)	<0.0001
Diuretics	326 (27.7)	1050 (17.9)	<0.0001
Drugs for acid related disorders	320 (27.2)	1097 (18.7)	<0.0001
Antiemetics and antinauseants	304 (25.9)	819 (13.9)	<0.0001
Drugs used in diabetes	287 (24.4)	624 (10.6)	<0.0001
Muscle relaxants	277 (23.6)	839 (14.3)	<0.0001
Contrast media	257 (21.9)	89 (1.5)	<0.0001
Anti-epileptics	251 (21.4)	629 (10. 7)	<0.0001
Beta blocking agents	246 (20.9)	827 (14.1)	<0.0001
Calcium channel blockers	196 (16.7)	674 (11.5)	<0.0001
Antihistamines for systemic use	195 (16.6)	662 (11.3)	<0.0001
Urologicals	190 (16.2)	682 (11.6)	<0.0001
Vitamins	188 (16.0)	435 (7.4)	<0.0001
Antivirals for systemic use	157 (13.4)	594 (10.1)	0.0012
Ophthalmologicals	157 (13.4)	576 (9.8)	0.0003
Drugs for obstructive airway diseases	132 (11.2)	441 (7.5)	<0.0001
Pituitary and hypothalamic hormones and analogs	130 (11.1)	15 (0.3)	<0.0001
Anesthetics	129 (11.0)	298 (5.1)	<0.0001
Antibiotics and chemotherapeutics for dermatological use	127 (10.8)	406 (6.9)	<0.0001
Antithrombotic agents	118 (10.0)	406 (6.9)	0.0002
Drugs for functional gastrointestinal disorders	115 (9.8)	291 (5.0)	<0.0001

Supplementary Table 2 Prevalence of all concomitant medications by ingredient

		Acromegaly cohort (N=1,175)	Control cohort (N=5,875)	
Ingredient, n (% of patients)	Form			p-value
Acetaminophen	Oral	541 (46.0)	1824 (31.0)	<0.0001
Amoxicillin	Oral	457 (38.9)	1616 (27.5)	<0.0001
Hydrocodone	Oral	428 (36.4)	1445 (24.6)	<0.0001
Levothyroxine	Oral	400 (34.0)	514 (8.7)	<0.0001
Azithromycin	Neither oral nor injection	304 (25.9)	1262 (21.5)	0.0011
Clavulanate	Oral	282 (24.0)	862 (14.7)	<0.0001
Prednisone	Oral	263 (22.4)	938 (16.0)	<0.0001
Oxycodone	Oral	251 (21.4)	695 (11.8)	<0.0001
Fluticasone	Neither oral nor injection	246 (20.9)	938 (16.0)	<0.0001
Ciprofloxacin	Oral	236 (20.1)	692 (11.8)	<0.0001
Gadobutrol	Injection	236 (20.1)	77 (1.3)	<0.0001
Hydrochlorothiazide	Oral	235 (20.0)	804 (13.7)	<0.0001
Metformin	Oral	234 (19.9)	514 (8.7)	<0.0001
Potassium chloride	Oral	215 (18.3)	497 (8.5)	<0.0001
Dexamethasone	Neither oral nor injection	213 (18.1)	744 (12.7)	<0.0001
Hydrocortisone	Oral	211 (18.0)	4 (0.1)	<0.0001
Triamcinolone	Neither oral nor injection	210 (17.9)	815 (13.9)	0.0005
Fentanyl	Neither oral nor injection	193 (16.4)	495 (8.4)	<0.0001
Ondansetron	Injection	192 (16.3)	491 (8.4)	<0.0001
Ondansetron	Oral	187 (15.9)	445 (7.6)	<0.0001
Cephalexin	Oral	185 (15.7)	675 (11.5)	0.0001
Polyethylene glycol 3350	Oral	181 (15.4)	367 (6.2)	<0.0001
Cyclobenzaprine	Oral	180 (15.3)	559 (9.5)	<0.0001
Ibuprofen	Oral	178 (15.1)	704 (12.0)	0.0032
Midazolam	Injection	177 (15.1)	488 (8.3)	<0.0001
Testosterone	Neither oral nor injection	177 (15.1)	73 (1.2)	<0.0001
Albuterol	Neither oral nor injection	177 (15.1)	743 (12.6)	0.0280
Gadopentetate dimeglumine	Injection	177 (15.1)	55 (0.9)	<0.0001
Atorvastatin	Oral	176 (15.0)	685 (11.7)	0.0018
Trimethoprim	Oral	175 (14.9)	566 (9.6)	<0.0001
Sulfamethoxazole	Oral	174 (14.8)	561 (9.5)	<0.0001
Lisinopril	Oral	173 (14.7)	754 (12.8)	0.0887
Tramadol	Oral	170 (14.5)	497 (8.5)	<0.0001
Sodium chloride	Injection	170 (14.5)	419 (7.1)	<0.0001
Doxycycline	Oral	170 (14.5)	592 (10.1)	<0.0001

		Acromegaly cohort (N=1,175)	Control cohort (N=5,875)	
**Ingredient, n (% of patients)**	**Form**			**p-value**
Codeine	Oral	168 (14.3)	692 (11.8)	0.0183
Methylprednisolone	Neither oral nor injection	167 (14.2)	683 (11.6)	0.0148
Omeprazole	Oral	162 (13.8)	555 (9.4)	<0.0001
Amlodipine	Oral	161 (13.7)	544 (9.3)	<0.0001
Sodium chloride	Oral	161 (13.7)	328 (5.6)	<0.0001
Methylprednisolone	Injection	141 (12.0)	557 (9.5)	0.0097
Gabapentin	Oral	140 (11.9)	351 (6.0)	<0.0001
Lidocaine	Neither oral nor injection	137 (11.7)	304 (5.2)	<0.0001
Levofloxacin	Oral	137 (11.7)	420 (7.1)	<0.0001
Ketorolac	Injection	134 (11.4)	420 (7.1)	<0.0001
Ergocalciferol	Oral	132 (11.2)	295 (5.0)	<0.0001
Simvastatin	Oral	132 (11.2)	512 (8.7)	0.0073
Promethazine	Oral	129 (11.0)	473 (8.1)	0.0013
Meloxicam	Oral	126 (10.7)	477 (8.1)	0.0043
Sodium bicarbonate	Oral	124 (10.6)	240 (4.1)	<0.0001
Metoprolol	Oral	124 (10.6)	454 (7.7)	0.0016
Testosterone	Injection	121 (10.3)	57 (1.0)	<0.0001
Alprazolam	Oral	119 (10.1)	320 (5.4)	<0.0001

Supplementary Table 3 Number of medication ingredients by comorbidity

	Oral medications			Injectable medications		
**Comorbidity**<br>**Number of different ingredients, n (% of patients)**			**p-value***			**p-value***
**Cardiovascular disorder**	**Acromegaly Cohort (N=794)**	**Control Cohort (N=2,844)**		**Acromegaly Cohort (N=794)**	**Control Cohort (N=2,844)**	
0	14 (1.8)	118 (4.1)	0.0021	191 (24.1)	1244(43.7)	<0.0001
1	18 (2.3)	103 (3.6)	0.0767	147 (18.5)	480 (16.9)	0.3048
2–3	55 (6.9)	311 (10.9)	0.0011	170 (21.4)	519 (18.2)	0.0501
>3	707 (89.0)	2312 (81.3)	<0.0001	286 (36.0)	601 (21.1)	<0.0001
**Type 2 diabetes and hyperglycemia**	**Acromegaly Cohort (N=223)**	**Control Cohort (N=524)**		**Acromegaly Cohort (N=233)**	**Control Cohort (N=524)**	
0	1 (0.45)	10 (1.9)	0.2364	24 (10.8)	149 (28.4)	<0.0001
1	3 (1.3)	6 (1.1)	0.8911	38 (17.0)	90 (17.2)	0.9512
2–3	11 (4.9)	29 (5.5)	0.8755	59 (26.5)	120 (22.9)	0.3429
>3	208 (93.3)	479 (91.4)	0.4780	102 (45.7)	165 (31.5)	0.0003
**Sleep apnea**	**Acromegaly Cohort (N=293)**	**Control Cohort (N=460)**		**Acromegaly Cohort (N=293)**	**Control Cohort (N=460)**	
0	5 (1.7)	9 (2.0)	0.9768	45 (15.4)	154 (33.5)	<0.0001
1	6 (2.0)	16 (3.5)	0.3605	46 (15.7)	74 (16.1)	0.9685
2–3	23 (7.8)	34 (7.4)	0.9278	63 (21.5)	100 (21.7)	0.9891
>3	259 (88.4)	401 (87.2)	0.7015	139 (47.4)	132 (28.7)	<0.0001
**Arthritis and musculoskeletal disorders**	**Acromegaly Cohort (N=234)**	**Control Cohort (N=748)**		**Acromegaly Cohort (N=234)**	**Control Cohort (N=748)**	
0	5 (2.1)	20 (2.7)	0.8279	33 (14.1)	177 (23.7)	0.0025
1	2 (0.85)	23 (3.1)	0.1002	33 (14.1)	134 (17.9)	0.2095
2–3	9 (3.8)	51 (6.8)	0.1335	46 (19.7)	190 (25.4)	0.0879
>3	218 (93.2)	654 (87.4)	0.0211	122 (52.1)	247 (33.0)	<0.0001

	Oral medications			Injectable medications		
**Comorbidity**<br>**Number of different ingredients, n (% of patients)**			**p-value***			**p-value***
**Bone disorder**	**Acromegaly Cohort (N=96)**	**Control Cohort (N=218)**		**Acromegaly Cohort (N=96)**	**Control Cohort (N=218)**	
0	0 (0.00)	9 (4.1)	0.0983	15 (15.6)	65 (29.8)	0.0118
1	3 (3.1)	1 (0.46)	0.1631	15 (15.6)	46 (21.1)	0.3295
2–3	2 (2. 1)	12 (5.5)	0.2907	20 (20.8)	52 (23.9)	0.6594
>3	91 (94.8)	196 (89.9)	0.2287	46 (47.9)	55 (25.2)	0.0001
**Malignancy**	**Acromegaly Cohort (N=265)**	**Control Cohort (N=505)**		**Acromegaly Cohort (N=265)**	**Control Cohort (N=505)**	
0	4 (1.5)	16 (3.2)	0.2557	47 (17.7)	166 (32.9)	<0.0001
1	5 (1.9)	17 (3.4)	0.3456	50 (18.9)	88 (17.4)	0.6915
2–3	17 (6.4)	37 (7.3)	0.7474	51 (19.2)	105 (20.8)	0.6796
>3	239 (90.2)	435 (86.1)	0.1332	117 (44.2)	146 (28.9)	<0.0001
**Hypopituitarism and disorder of hypothalamus**	**Acromegaly Cohort (N=309)**	**Control Cohort (N=10)**		**Acromegaly Cohort (N=309)**	**Control Cohort (N=10)**	
0	7 (2.3)	0 (0.0)	0.5383	61 (19.7)	2 (20.0)	0.7015
1	10 (3.2)	0 (0.0)	0.7309	57 (18.4)	3 (30.0)	0.6107
2–3	25 (8.1)	1 (10.0)	0.7114	72 (23.3)	2 (20.0)	0.8909
>3	267 (86.4)	9 (90.0)	0.8863	119 (38.5)	3 (30.0)	0.8302

*p-values calculated using an unpaired t-test.
